# Whole genome analysis of rare deleterious variants adds further evidence to BRSK2 and other risk genes in Autism Spectrum Disorder

**DOI:** 10.21203/rs.3.rs-3468592/v1

**Published:** 2023-10-28

**Authors:** Elena Bacchelli, Marta Viggiano, Fabiola Ceroni, Paola Visconti, Annio Posar, Maria Scaduto, Laura Sandoni, Irene Baravelli, Cinzia Cameli, Magali Rochat, Alessandra Maresca, Alessandro Vaisfeld, Davide Gentilini, Luciano Calzari, Valerio Carelli, Michael Zody, Elena Maestrini

**Affiliations:** University of Bologna; University of Bologna; University of Bologna; IRCCS Istituto delle Scienze Neurologiche di Bologna; IRCCS Istituto delle Scienze Neurologiche di Bologna; IRCCS Istituto delle Scienze Neurologiche di Bologna; University of Bologna; University of Bologna; University of Bologna; IRCCS Istituto delle Scienze Neurologiche di Bologna; IRCCS Istituto delle Scienze Neurologiche di Bologna; University of Bologna; University of Pavia; Istituto Auxologico Italiano IRCCS; IRCCS Istituto delle Scienze Neurologiche di Bologna; New York Genome Center (NYGC); University of Bologna

## Abstract

Autism spectrum disorder (ASD) is a complex neurodevelopmental condition with a strong genetic component in which rare variants contribute significantly to risk. We have performed whole genome and/or exome sequencing (WGS and WES) and SNP-array analysis to identify both rare sequence and copy number variants (SNVs and CNVs) in 435 individuals from 116 ASD families. We identified 37 rare potentially damaging de novo SNVs (pdSNVs) in cases (n = 144). Interestingly, two of them (one stop-gain and one missense variant) occurred in the same gene, BRSK2. Moreover, the identification of 9 severe de novo pdSNVs in genes not previously implicated in ASD (*RASAL2, RAP1A, IRX5, SLC9A1, AGPAT3, MGAT3, RAB8B, MGAT5B, YME1L1*), highlighted novel candidates. Potentially damaging CNVs (pdCNVs) provided support to the involvement of inherited variants in *PHF3, NEGR1, TIAM1* and *HOMER1* in neurodevelopmental disorders (NDD), although mostly acting as susceptibility factors with incomplete penetrance. Interpretation of identified pdSNVs/pdCNVs according to the ACMG guidelines led to a molecular diagnosis in 19/144 cases, but this figure represents a lower limit and is expected to increase thanks to further clarification of the role of likely pathogenic variants in new ASD/NDD candidates.

In conclusion, our study strengthens the role of *BRSK2* and other neurodevelopmental genes in ASD risk, highlights novel candidates and contributes to characterize the allelic diversity, mode of inheritance and phenotypic impact of *de novo* and inherited risk variants in ASD/NDD genes.

## Introduction

Autism spectrum disorder (ASD) is a neurodevelopmental condition characterized by social and communication difficulties, repetitive behaviours and unusually restricted or stereotyped interests^1^. ASD is both clinically and genetically heterogenous. Its architecture is characterized by a complex interplay between three major categories of genetic risk: common polygenic variation, rare inherited and *de novo* mutations. The contribution of each component varies between individuals. At one extreme, the susceptibility is mainly attributable to the polygenic risk determined by thousands of common risk alleles, each exerting a small additive effect^2^. At the other extreme, *de novo* variants (DNVs) can act as major contributors, leading in some cases to almost monogenic conditions. High-impact DNVs are estimated to affect at least 10% of ASD cases, but their contribution varies significantly according to the ascertainment strategy of the studied cohort, with a higher burden in ASD cases with comorbid intellectual disability (ID) and developmental disorders (DD)^3–6^.

Given the large effect size of individual pathogenic DNVs, exome/genome sequencing studies (WES/WGS) of large cohorts of ASD families have led to a considerable progress in gene discovery, identifying hundreds of high-confidence genes involved in ASD or other neurodevelopmental disorders (NDDs) susceptibility^4–6^.

The contribution of rare inherited variants has proven to be more difficult to be characterised. However, large family studies have recently managed to identify genes where the risk is mostly driven by rare loss-of-function (LoF) inherited variants, supporting the idea that by increasing the number of autism cases additional moderate-risk genes will be identified^7,8^.

Early microarray studies have also significantly enhanced our understanding of the genetic landscape of ASD and other NDDs, pinpointing dozens of copy number variation (CNV) regions and dosage-sensitive genes^9,10^. A recent study showed that 10.5% of NDD individuals carry CNVs of potential clinical relevance, of which about 40% are recurrent CNVs triggered by flanking repetitive sequences (recurrent genomic disorder, RGD), and > 50% are CNVs disrupting one or more genes already implicated in NDDs^11^. The overall resolution of CNV studies is now increasing thanks to WGS: this approach enables the discovery of previously undetected structural variations (small CNVs, CNVs in complex genomic regions and complex events), and strongly improves the ability to define the breakpoints, which is crucial for variant interpretation. WGS is thus standing out among less comprehensive technologies, as all sizes and types of variation are detectable with base-pair resolution in a single test^6^.

Despite these remarkable advances, many ASD cases remain genetically unexplained, highlighting a continuing need for further discovery efforts. In this context, family-based sequencing studies still represent a key approach for the identification of *de novo* variants with large effect that can provide new insights on risk genes, variant types and molecular mechanisms underlying the disorder, potentially offering promising targets for translational research.

Here, we present an integrated analysis of different classes of variants identified through WGS/WES and SNP-array in a cohort of 116 ASD multiplex and simplex families. This approach allowed us to characterize the contribution of rare *de novo* and inherited coding SNVs, indels and CNVs in our sample and to assess their phenotypic impact by exploring the presence of comorbidities in individuals carrying such variants.

## Results

### Overview of the cohort

Here we report the genomic characterisation of 435 individuals from 116 ASD families, comprising 144 individuals with ASD, 6 siblings with specific learning disabilities (SLD), 55 unaffected siblings, and 230 parents. Among the 144 affected individuals, 89 were from simplex families, while 55 belonged to 27 multiplex families. DNA samples were available for both parents for 114/116 families. Among the multiplex families, 22 included two affected siblings, two included monozygotic twin pairs, one family included three affected siblings, while in two families the affected individuals were a child and a paternal uncle.

Phenotypic data of ASD individuals are reported in Table S1 and Fig. S1. Mild to severe ID was present in 56% of probands, with 16/144 cases with severe ID. The vast majority of probands had language problems (98.6%) and the rate of females with absent speech was significantly higher than in males (16/34 vs 30/110, two-sided chi2 *p*-value = 0.03).

The score distribution of Social and Communication Disorders Checklist (SCDC)^12^ in the entire cohort and of The Broad Autism Phenotype Questionnaire (BAPQ)^13^ in parents are shown in Fig. S2.

Genetic data consisted of Illumina Infinium PsychArray genotyping for all families, WGS of 105 families and WES of 29 families (Fig. S3).

MDS analysis was performed for ancestry determination, anchoring our cohort data to the 1000 Genomes Project. We visually inspected the first two MDS coordinates and found no discrepancy between genotype-computed and self-reported ancestry. Individuals of non-European ancestry comprise ~15% of our sample (66 individuals from 19 families, for a total of 26 cases), including one African, one South Asian and 17 Admixed families (Fig. S4).

### Rare coding sequence variant analysis

We analysed WES and WGS data from all 435 individuals of our cohort, focussing on rare variants affecting coding exons and canonical splice sites as these provide the most direct links between gene function and disease pathogenesis. We did not use WGS data to investigate mitochondrial DNA, as deep sequencing of the entire mitogenome and quantification of mtDNA cellular content of this cohort has been previously described^14^.

#### De novo variants

**Figure 1:**
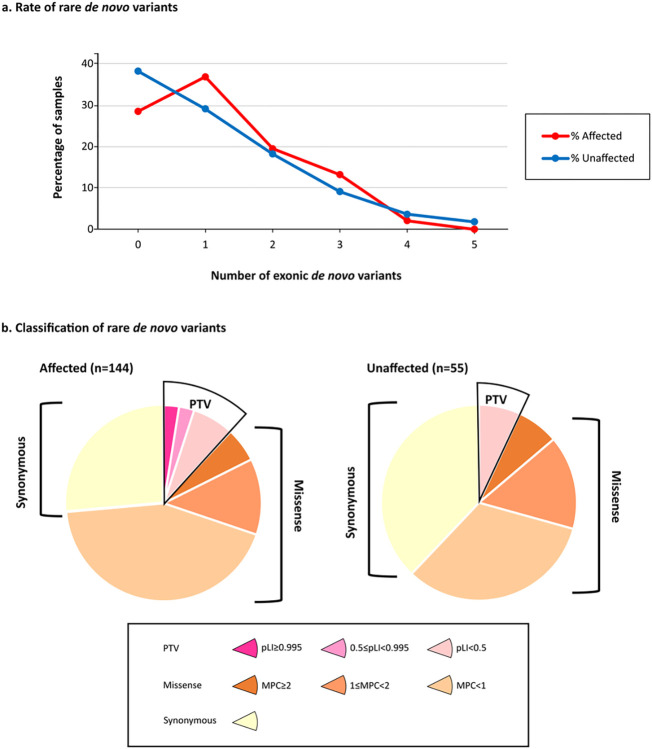
Rare *de novo* coding variants in cases and unaffected siblings. (a) Rare *de novo* coding variants in cases and unaffected siblings. (a) Rare coding de novo variants per individual in our cohort (ASD cases=144, unaffected siblings=55). (b) Distribution of rare de novo coding variants in cases and unaffected siblings: the pie charts represent rare de novo coding variants split by predicted functional consequences, represented by different colours. PTVs and missense variants are divided into three tiers of predicted functional severity, represented by different shade, based on the pLI (0.995–1, 0.5–0.995, 0–0.5) and MPC metrics (MPC ≥2 (DmisB), 2> MPC ≥1 (DmisA), 1> MPC ≥0), respectively.

We identified a total of 243 rare DNVs in protein-coding exons (MAF ≤ 0.1% in reference databases): 178 in 144 ASD individuals and 65 in 55 unaffected siblings (Fig. 1a).

The number of DNVs per child was consistent with the rate reported in other studies^4^ and similar between individuals with autism and their siblings (mean rate of 1.24 and 1.18, respectively). The percentage of cases and unaffected siblings carrying at least one rare *de novo* SNV (cases: 103/144, 71.5%; unaffected siblings: 35/55, 63%; two-sided chi2 *p*-value = 0.28) was also comparable to previous studies.

Then we catalogued rare DNVs in seven bins of predicted functional severity: three bins for PTVs according to pLI score^15,16^, three bins for missense variants based on the MPC score^17^, and a single bin for synonymous variants. The variants predicted to be more deleterious account for 24% of the DNVs found in cases: 5% were PTVs in constrained genes (pLI ≥ 0.5), hereafter referred to as “PTV_0.5_”, and 18% were Dmis (damaging missense variants with MPC ≥ 1). The remaining DNVs were missense with MPC < 1 (43%), synonymous (26%), and PTVs in unconstrained genes (7%), consistently with the previously reported bin distribution in a family sample of 6,430 ASD cases^4^. Interestingly, no PTV_0.5_ were identified in unaffected siblings, supporting a larger effect on liability of this class of variants (Fig. 1b, Table S2).

Among the 37 rare *de novo* PTV_0.5_ and Dmis identified in our cases, hereafter defined as potentially damaging SNVs (pdSNVs), two (one PTV and one DmisB) occurred in *BRSK2* in two different families (Table 1). Beyond these *de novo* pdSNVs, we also identified a stop-gain variant of unknow origin in *SHANK3* in the female proband of simplex family 123 (maternal DNA was unavailable). However, since LoF variants in *SHANK3* usually arise *de novo*^8^, this was deemed as likely *de novo* (Table 1).

STRING enrichment analysis of the 36 genes hosting the 37 *de novo* pdSNVs in probands detected a significant enrichment in gene interactions (12 vs 5 expected edges, 2.4-fold enrichment, *p*-value = 0.00318, one-tailed hypergeometric test), while no significant interaction enrichment was identified for 40 genes hosting 42 synonymous de novo variants in probands (4 vs 3 expected edges, *p*-value = 0.456).

If we restricted the STRING analysis to the 18 genes hosting the 19 most severe *de novo* pdSNVs (8 PTV_0.5_ and 11 DmisB), there was still a significant interaction enrichment (5 vs 1 expected edges, *p*-value = 0.0184). Gene Ontology (GO) enrichment analysis of these 18 genes identified 10 genes in the “regulation of transport” category (GO:0051049, 6.48-fold enrichment, FDR = 3.97×10^−3^), and 11 in the “regulation of localization” category (GO:0032879, 5.98-fold enrichment, FDR = 3.27×10^−3^), while no biological process resulted to be enriched for the 40 genes with de novo synonymous variants (Table S3).

#### Overall analysis of de novo and inherited variants

We next assessed the rate of *de novo* and inherited pdSNVs in cases and unaffected siblings and found no overall excess of such variants in cases (Fig. S5).

**Figure 2:**
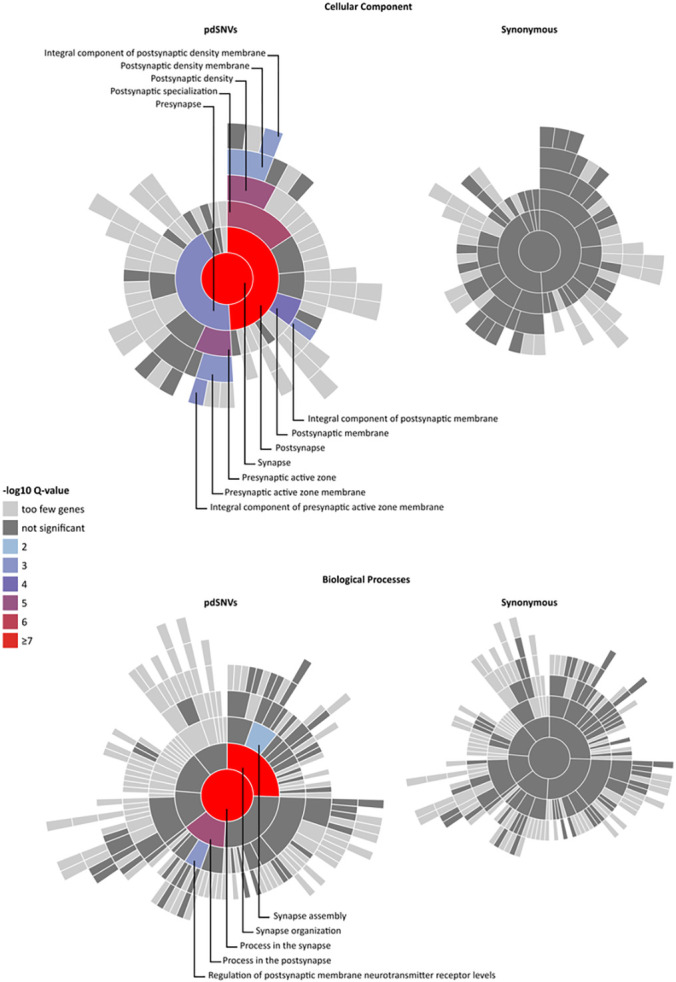
Enrichment for *de novo* and inherited pdSNVs in SynGO Genes. Visualisation of gene set enrichment analyses (GSEA) of genes harbouring pdSNVs (left) and synonymous variants (right) in affected individuals, each compared to a background set of brain-expressed genes. All Cell Components (CC) or Biological Process (BP) related terms with gene annotations in SynGO are plotted in a circular fashion, with the highest hierarchical term (“synapse” for CC or “process in synapse” for BP) in the centre and each layer of subclasses in outward concentric rings. Over-represented synaptic terms are indicated with different colours, according to the Q-value, and are reported in detail in Table S5. The CC and BP plots of genes affected by rare pdSNVs (left) show an enrichment of synaptic terms, while no enrichment emerged from the genes hosting rare synonymous SNVs (right).

Given the well-known role of synaptic genes in ASD pathogenesis, we used the SynGO platform^18^ (dataset version: 20210225) to investigate whether the affected individuals showed an enrichment of rare pdSNVs in genes involved in synaptic components or functions. Among the 2,150 genes harbouring pdSNVs in cases (Table S4), 251 were SynGO annotated genes. When compared with the “brain expressed” background set (18,035 unique genes including 1,225 SynGO annotated genes), our list showed a significant enrichment at 1% FDR for 12 Cellular Component terms and 5 Biological Processes (Fig. 2, Table S5). In contrast, the same analysis on the 6,666 genes carrying rare synonymous variants in ASD individuals highlighted 517 SynGO annotated genes, without any significant enrichment for Cellular Component or Biological Processes terms (Fig. 2).

**Figure 3:**
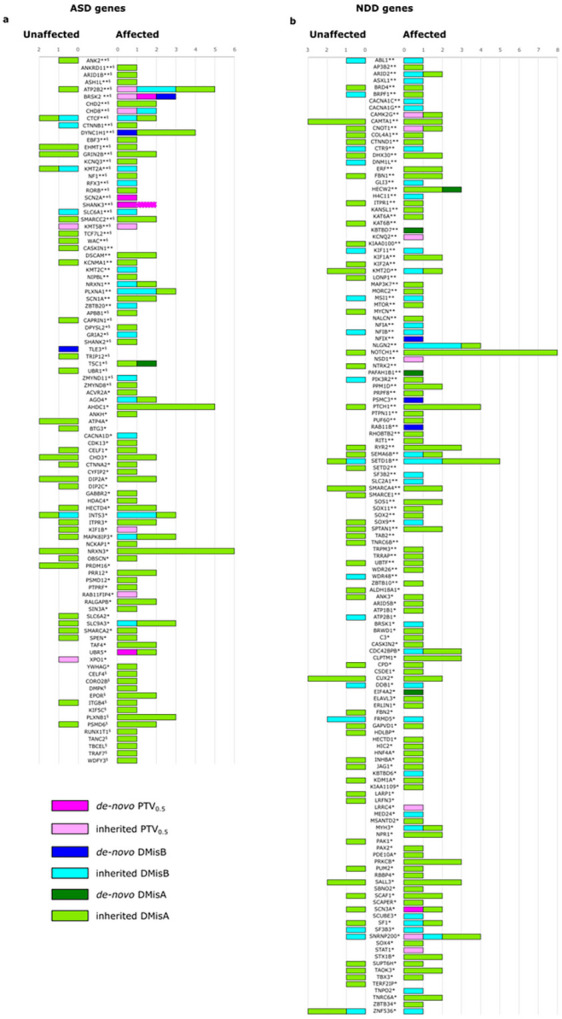
Contribution of *de novo* and inherited pdSNVs to high confidence ASD/NDD genes. *De novo* and inherited pdSNVs include PTVs in genes with pLI score ≥0.5 (PTV_0.5_), missense variants with MPC score ≥2 (DmisB) and missense variants with MPC score 1–2 (DmisA). Contribution of each variant type identified in ASD individuals and unaffected siblings for a list of genes previously associated to ASD (a) and NDD (b). The list of ASD genes comprised 185 genes associated at FDR ≤0.05^5^ and 135 genes with FDR <0.1^6^ (88 of which were common between the two lists). In our cohort, pdSNVs were identified in 98 ASD genes (a). The list of NDD genes included 452 genes from a list of 664 genes associated at FDR ≤0.05, after the exclusion of the genes already included among the 232 ASD genes^5^. In our cohort, pdSNVs were identified in 141 NDD genes (b). **, genes with FDR ≤0.001^5^; *, genes with FDR ≤0.05^5^; §, genes with FDR <0.1^6^; dotted line indicates a putative *de novo* PTV_0.5_.

To assess the contribution of deleterious variants in high-confidence ASD and/or NDD genes (n = 684, Table S6)^5,6^, we selected all the *de novo*/inherited pdSNVs located in such genes. Our study identified rare pdSNVs in 98/232 high-confidence ASD genes (Fig. 3a) and in 141/452 high-confidence NDD genes (Fig. 3b).

While the rate of inherited rare variants in the 684 ASD/NDD genes was similar between cases and unaffected siblings, we observed an increased rate of *de novo* variants in affected individuals (16/144 cases (11.1%) vs 1/55 unaffected sibs (1.8%), Fisher’s exact test *p*-value = 0.04,). Interestingly, probands carrying *de novo* pdSNVs in these genes versus those who did not, showed a significant positive association with severe ID (non-verbal IQ < 35) (two-sided chi2 *p*-value = 0.0066, OR = 4.83) (Table S7). When considering only the most severe *de novo*/inherited pdSNVs (21 PTV_0.5_ and 63 DmisB), 64 cases (44%) had at least one variant in these genes (17 probands had more than one variant). Comparing the probands with and without severe pdSNVs, we observed a significant association with severe ID (Fisher’s Exact test *p*-value = 0.014, OR = 4.25) and epilepsy (Fisher’s Exact test *p*-value = 0.03, OR = 4.2) (Table S7).

### Rare copy number variant analysis

Discovery of rare CNVs was performed by integrating CNV calls from SNP-array data on the entire collection of families with those from WGS of 105 families. After filtering, we defined a high-confidence set of 192 rare (frequency < 1% in our dataset) genic CNVs in cases and SLD siblings (Table S8). These included 93 CNVs identified by both SNP-array and WGS, 79 detected only by WGS and 20 identified only by SNP-array in families not analysed by WGS. Among variants detected only by WGS, 32 (40.5%) were deletions (median size = 20.2 kb) and 47 (59.5%) duplications (median size = 31.7 kb).

We prioritised four categories of potentially damaging CNVs (pdCNVs) (Table 2):

### Multiple hits in families with CNVs in genomic disorders loci

Since CNVs in RGD loci are often inherited and require secondary hits to reach the liability threshold for disease, we checked whether the probands heterozygotes for these CNVs also had *de novo* pdSNVs, PTV_0.5_ in NDD genes or pdCNVs inherited from the parent not transmitting the recurrent CNV. Four families carried additional variants of interest (Fig. S7). In Fam81, the proband had a likely causative *de novo* DmisB variant in *NFIX*, which allowed us to redefine his phenotype as Malan syndrome^21^, while supporting the ACMG classification of 15q13.3 duplications as VUS. In Fam117, both affected children inherited a paternal 15q11.2 deletion and a maternal exonic deletion of *PHF3*. Moreover, each of them had a *de novo* Dmis, one in *YME1L1* and the other in *RCCD1* (Table 1, Fig. S7). Interestingly, their father exhibited autistic traits: according to the SCDC test^12,22^, he had social and communication difficulties (SCDC score = 15, an outlier in the SCDC parents’ score distribution) (Fig. S2b), while in the BAPQ^13^ he exhibited impairments in the pragmatic language domain.

### Autosomal and X-linked recessive events

To identify variants potentially acting with a recessive inheritance, we looked for homozygous and compound heterozygous pdSNVs/pdCNVs.

Biallelic pdSNVs events were identified in six genes (Table S9): five harboured biallelic inherited DmisA, while *DYNC1H1* harboured a maternal DmisA and a *de novo* DmisB. However, *DYNC1H1* has been reported to act through a dominant mode of inheritance, therefore the *de novo* DmisB is likely to be the main causative variant for the ASD phenotype in this individual^23^.

A compound pdCNV-pdSNV event was identified in Fam91, where proband 91.3 carries a 412 kb deletion of unknown origin (paternal DNA was unavailable) and a maternal DmisA in the remaining allele of *PAX7*, a gene labelled as having a biallelic mode of inheritance in Genomics England neurology and NDD panel.

To identify potentially causative X-linked events, we searched for hemizygous pdSNVs and pdCNVs present in male probands and absent in unaffected brother(s) (Table S11). We identified 4 DmisB and 28 DmisA: 16 of these are absent in males in gnomAD (v2.1.1/v3.1.2), 12 of which map in GeneTrek NDD genes.

### Polygenic risk scores

To analyse the contribution of common genetic variants to ASD risk, we calculated PRS from our sample using summary statistics from a recent ASD GWAS^2^. To test technical reproducibility, we compared PRS in two MZ twin pairs, and no between-twin difference was detected. Even if the small difference in mean PRS between cases and unaffected sibs was not significant (Fig. S8a), we observed a significant PRS over-transmission in cases (n = 119, pTDT mean = 0.222, *p*-value = 0.019), but not in unaffected siblings (n = 49, mean = 0.107, *p*-value = 0.45) (Fig. S8b).

## Discussion

We report an integrated analysis of rare protein-coding SNVs, indels and CNVs from WGS/WES and SNP-array data of a cohort of 116 ASD families. This analysis led to the identification of potentially damaging de novo and inherited variants expanding the allelic diversity and the mode of inheritance of specific ASD/NDD risk genes, while helping characterise their phenotypic impact. Moreover, the identification of *de novo* pdSNVs of high functional severity (PTV_0.5_ and DMisB) in 9 genes (*RASAL2, RAP1A, IRX5, SLC9A1, AGPAT3 MGAT3, RAB8B, MGAT5B, YME1L1*) not previously described as known high-confidence ASD/NDD genes^5,6,20^ highlighted novel candidates (Table 1). Among these, *RAB8B, RAP1A* and *SLC9A1* were also listed among the genes driving significantly enriched biological processes “regulation of transport” and “regulation of localization” in our GO enrichment analysis (Table S3).

**Figure 4:**
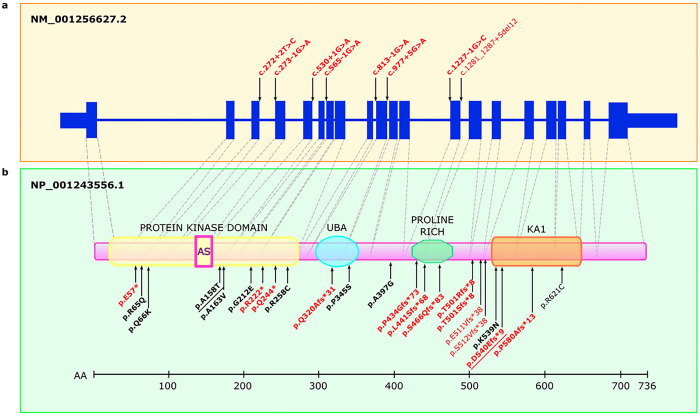
*BRSK2exons*, domains and reported variants. Schematic representation of the *BRSK2* gene structure (a) and protein domains (b), illustrating potentially damaging variants reported in this and previous studies. Protein domains include protein kinase domain (containing the active site, AS), ubiquitin-associated domain (UBA), proline-rich domain (Pro-Rich), and kinase-associated 1 (KA1) domain. Splice variants are shown above the schematic representation of the MANE transcript (upper panel), and protein-altering variants are shown below the schematic representation of BRSK2 (PTVs in red, missense variants in black). Confirmed *de novo* variants are highlighted in bold. The two variants identified in this study (p.(Ala158Thr), p.(Asp540GlufsTer9)) are underlined.

Among the 37 de novo pdSNVs detected in affected individuals, 15 were in high-confidence ASD/NDD genes^5,6^. Interestingly, two of them (one PTV and a DmisB) occurred in the same gene, *BRSK2*. The identification of two *de novo* events in *BRSK2* in two different families is noteworthy given that *BRSK2* is highly constrained with only 29 *de novo* missense and PTV variants reported to date^5,6,8,24–28^ (Fig. 4). The frameshift variant is novel, while the DmisB variant has been reported in gnomAD v3.1.2 in one individual recruited as a case in a neurologic/psychiatric study (gnomAD “neuro” dataset). Intriguingly, large sequencing studies comparing the genetic architecture of ASD with other NDDs to discriminate between genes predominantly underlying ASD and those affecting development more broadly have found evidence for *BRSK2* only from ASD cohorts^5^. Therefore, a possible explanation for the high frequency of *BRSK2* rare *de novo* mutations in our study is that our cohort was specifically ascertained for ASD. Moreover, the two probands with *BRSK2 de novo* pdSNVs have both idiopathic ASD and no comorbidities (Supplementary Information). Taken together, these data support the hypothesis that *BRSK2* belongs to a set of genes that, when disrupted, alter the core features of ASD and thus are particularly promising for neurobiological studies of ASD. *BRSK2* encodes for a serine/threonine-protein kinase involved in axonogenesis and polarization of cortical neurons. Its role in neurodevelopment has been studied in mice models^29^ and, most recently, in a brsk2-deficient zebrafish model that showed ASD-like behaviours^30^.

Another *de novo* pdSNV of particular interest is the stop-gain variant identified in *SCN3A*, a gene predicted to be highly intolerant to LoF. This variant occurs in the last exon and while it is expected to escape nonsense-mediated decay (NMD), it is predicted to cause the loss of 371 aa involving part of the fourth transmembrane domain (177 aa) and the entire cytoplasmic C-terminal tail (Fig. S9). Pathogenic variants in this gene lead to a spectrum of neurodevelopmental conditions including epilepsy, as well as developmental brain malformations but, to our knowledge, no PTVs in *SCN3A* have been previously reported in ASD individuals. The clinical description of proband 40.3 is reported in Supplementary Information.

In addition to expanding the spectrum of potentially damaging variants in recently implicated ASD/NDD genes, this study also helps clarify their mode of inheritance. An example is given by the heterozygous deletion disrupting *PHF3* identified in Fam117 (Table 2). This gene encodes a PHD finger protein that regulates transcription and mRNA stability and is involved in the timely expression of neuronal genes during neurogenesis^31^. *PHF3* is a high-confidence ASD gene (Table S6); its strong association with ASD (FDR < 0.1) was mainly driven by de novo PTVs^6^. Rare inherited PTVs in *PHF3* are also associated to ASD risk, as shown by Transmission Disequilibrium Test (TDT) in 13,000 ASD families^8^. Taken together, these data suggest that *PHF3* belongs to a subset of ASD genes increasing risk through both *de novo* and inherited PTVs. The maternal *PHF3* deletion identified in our cohort provides further support to the role of inherited deleterious variants in this gene. Notably, the inheritance pattern observed in our family suggests an incomplete penetrance, likely to be modulated by other risk factors. Indeed, in Fam117 we identified additional rare inherited and *de novo* risk variants that might contribute to the cognitive and behavioural phenotypes of the two affected children with the deletion (Fig. S7).

Our CNV analysis also provides additional support to inherited variants in the candidate-NDD gene *NEGR1*. We identified a maternally inherited intragenic deletion in the simplex family 108 (Table 2). Previous studies have reported similar *NEGR1* microdeletions, inherited or of unknown origin, in individuals with developmental delay, ID and autistic features^32,33^. *NEGR1* was also highlighted by the largest GWAS meta-analysis performed to date for ASD as the only protein-coding gene of the four genome-wide significant loci shared between ASD and major depression^2^. NEGR1 is a cell adhesion protein involved in neurite outgrowth regulation, dendritic arborization and synapse formation^34^. Interestingly, *Negr1* deficiency in mouse results in abnormal neuronal growth and migration, abnormal spine density during cortical development and impaired social behaviour^35,36^.

The identification of an inherited deletion disrupting *TIAM1* is also of interest (Table 2). This gene encodes a guanine nucleotide exchange factor (GEF) that regulates RAC1 signaling pathway, which affects neuronal morphogenesis and neurite outgrowth^37^. *TIAM1* has been recently implicated in NDDs by a study reporting biallelic missense variants in 5 individuals with ID, language delay and seizures. Functional studies of three of these variants showed only a partial LoF effect^37^. In contrast, our deletion is predicted to determine a total LoF and thus might have a severe impact, even if monoallelic. The intolerance of *TIAM1* to LoF events is supported by high pLI and pHaplo scores and by the absence of exonic deletions in gnomAD and DGV. Taken together, these data suggest that *TIAM1* LoF events may contribute to NDD risk but with a reduced penetrance.

Given the complex architecture of ASD, where a diverse spectrum of variants contributes to the susceptibility, even within the same individual, WGS represents the ideal approach for a comprehensive investigation of all sizes and types of variants underlying the risk.

Notably, 18.5% of pdCNVs (5/27 CNVs identified in 105 families characterized with both WGS and SNP-array) would not have been detected without WGS, highlighting the benefits of WGS for the detection of smaller CNVs. Among these, WGS identified an exonic deletion of 19 kb in *HOMER1* not found from SNP data as only two probes map inside the deletion (Table 2, Fig. S10). HOMER1 is a key component of the postsynaptic density (PSD), where it exerts an important scaffolding role, interacting with multiple targets, including SHANK proteins^38^. The proband, who has a moderate early-onset autism (ADOS-2 comparison score = 6) without cognitive impairment (LEITER-R = 102), shares the deletion with the unaffected sister, who do not present autistic features or cognitive impairment, suggesting that it may act as a susceptibility factor with incomplete penetrance.

The clinical relevance of *de novo* pdSNVs/pdCNVs and recurrent CNVs was interpreted according to the ACMG guidelines^39^. Six pdSNVs and 1 CNV were classified as pathogenic while 30 pdSNVs and 3 CNVs as likely pathogenic (Tables 1 and 2). Of these, 19 variants were in high-confidence ASD-NDD genes or RGD loci, providing a molecular diagnosis in 19/144 ASD cases. This diagnostic yield (13%) is consistent with previous estimates^3^, although most likely represents an underestimate of the true etiologic yield, since all probands were pre-screened by clinical aCGH and excluded if positive. Moreover, continuous discovery efforts and functional studies may help to clarify the role of the other 21 pathogenic/likely pathogenic variants, establishing their diagnostic relevance, increasing the current yield, and implicating new ASD risk genes. Notably, among the pathogenic variants, the *de novo* DmisB identified in exon 2 of *NFIX* prompted us to reassess the phenotype of the proband in Fam81, leading to a clinical diagnosis of Malan syndrome. This proband has early-onset severe autism, profound ID, dysmorphic features, macrocephaly and mild brain MRI anomalies, consistent with the main features of Malan syndrome^21,40^ (Supplementary Information).

Given the small size of our cohort, we have limited power in performing aggregate analysis of rare variants. Nevertheless, SynGO analysis of the rare *de novo* and inherited pdSNVs identified in affected individuals showed a significant enrichment in genes involved in synaptic components and processes, in contrast with the genes harbouring rare synonymous variants that did not display any synaptic enriched terms. Since synaptic dysregulation is widely recognized as an important component of ASD risk, this finding supports the pathological role of the pdSNVs identified in synaptic genes.

We also evaluated the frequency of pdSNVs in a list of high-confidence ASD/NDD genes, identifying a higher rate of rare *de novo* pdSNVs in the 144 cases compared to the 55 unaffected siblings (11% versus 1.8%). Moreover, probands carrying de novo pdSNVs were more likely to have severe ID and those with severe pdSNVs (regardless of inheritance status) were more likely to also have epilepsy. This finding aligns well with the observation that *de novo* variants are more frequently found in ASD cases with comorbidities and support a role for inherited more deleterious pdSNVs in cases with more severe phenotypes^41^.

WGS data were also used to compute PRS. The significant overtransmission of common risk variants from parents to ASD children is consistent with previous results^42^ and supports the additive role of common genetic variants in ASD susceptibility.

Our results should be interpreted in the context of the small sample size (116 families), which is the principal limitation of this study. Our cohort obviously is not sufficient to achieve statistical significance for the implication of new risk genes. However, our family-based study provides valuable new data on the allelic diversity and mutational mechanisms that impact specific genes contributing to ASD and NDDs, strengthening their involvement in these disorders and offering new insights into the genomic architecture underpinning ASD. Moreover, we identified potentially damaging variants in novel promising candidate genes that warrant further investigation.

## Methods

### Clinical assessment and description of samples

The cohort analysed in this study consisted of 116 ASD families recruited by “UOSI Disturbi dello Spettro Autistico” (IRCCS Istituto delle Scienze Neurologiche, Bologna, Italy).

This study was approved by the local Ethical Committee (Comitato Etico di Area Vasta Emilia Centro (CE-AVEC); code CE14060) and performed in accordance with the relevant regulations. All participants or substitute decision makers provided a written informed consent to participate to the study. For each family, we collected blood samples from all available family members, for a total of 435 individuals.

Clinical diagnoses were given by a team of clinicians according to the Diagnostic and Statistical Manual of Mental Disorders 5th edition (DSM-5)^43^. Specifically, ASD subjects were assessed using a set of standardized clinical tests to evaluate the presence and severity of ASD (Autism Diagnostic Observation Schedule-Second Edition, ADOS-2^44^ and The Childhood Autism Rating Scale-Second Edition, CARS-2^45^), to assess both developmental/cognitive levels (Bayley, PEP-3, Leiter-R, Griffith Scales, Wechsler Scales) and adaptive behaviour (Vineland Adaptive Behavior Scale, VABS)^46^ as well as discrete and clinical signs like mimicry, hyperactivity, sensory abnormalities and symptoms age onset^47^. Since measures of IQ were quantified using multiple methods, we also converted full-scale scores from different scales into five IQ categories (severe, moderate, mild, borderline and normal).

Family members were assessed for subclinical features using the Social and Communication Disorders Checklist (SCDC)^12^ and The Broad Autism Phenotype Questionnaire (BAPQ)^48,49^.

Electroencephalogram (EEG) and Magnetic Resonance Imaging (MRI) were also performed on 127 and 126 ASD individuals, respectively.

### Genotyping data processing

All genetic analyses were performed on DNA samples extracted from whole blood using a QIAamp DNA blood kit (Qiagen, Hilden, Germany).

The whole cohort (n = 435 individuals) was genotyped using the Illumina Infinium PsychArray-24 v1.3 BeadChip. Genotyping quality control was performed according to standard procedures^50^. Briefly, we excluded markers that exhibited high missingness rates (> 5%), low minor allele frequency (< 1%), or failed a test of Hardy–Weinberg equilibrium (p < 1 × 10^−5^). No individuals were excluded due to a high proportion of missing genotype data (≥ 2%), inconsistent sex information or for high rates of heterozygosity (> 3sd from the mean).

### Ancestry determination

SNP data were used to determine the ancestry of all individuals of our cohort with PLINK^51^. We first removed large-scale high-Linkage Disequilibrium (LD) regions and performed LD pruning using the option ‘--indep-pairwise 50 5 0.2’. Then, we performed genome-wide pairwise IBS calculations and multidimensional scaling (MDS) analysis, anchoring our cohort data to the data of the 1000 Genomes Project (20100804 release) (http://www.1000genomes.org/) to visualize genetic distances.

### Sequencing, quality control, variant calling and annotation

WGS was performed on 105 families, while WES was carried out for 29 families, with 18 families being analysed with both.

WES was performed using the NimbleGen SeqCap EZ MedExome enrichment kit (Roche) and Illumina NextSeq500 or HiSeq sequencers. All exomes had a read depth (DP) > 10x for 90% of the total exome coverage and > 20x for 80%. Data analysis was performed using CoVaCS^52^, a pipeline exploiting three different calling algorithms (GATK, Varscan and Freebayes) to generate a final set of high-confidence variants.

WGS was performed at the New York Genome Center. Alignment and post-processing were carried out using the standard pipeline for the CCDG^53^ and the GRCh38_full_analysis_set_plus_decoy_hla.fa reference genome. Briefly, raw reads were aligned to the GRCh38 reference genome (Burrows–Wheeler Aligner-MEM)^54^, duplicate reads were marked (Picard v.2.4.1, http://broadinstitute.github.io/picard/), base scores were recalibrated and indels were realigned (GATK v.3.5.0)^55^. SNVs and indels were called using the GATK HaplotypeCaller and FreeBayes v1.1.0 (https://github.com/ekg/freebayes) on a per-family basis. After some basic sample-level filtering (DP > 9 for parents and children, child genotype quality (GQ) > 20, child allele balance (AB) > 0.25, parent alternate allele count (AO) = 0), *de novo* variants were retained only if they had been identified by both GATK and Freebayes.

Variant annotation was performed with ANNOVAR^56^, using RefSeq for gene-based annotation (Genome Build hg38). Annotated variants were filtered in order to retain only coding and splicing variants, and to remove low-quality variants (Coverage (DP) < 10, Genome Quality (GQ) < 20 and Variant Quality Score Recalibration (VQSR) not indicating PASS). We also removed variants located in regions known to be difficult for variant calling (HLA, mucins, and olfactory receptors).

Rare variants were defined according to the population allele frequencies in the non-neurological subset of gnomAD v.2.1.1 and the entire dataset of gnomAD v.3.0; MAF thresholds of ≤ 0.1% and ≤ 1% were applied to analyse variants according to a monoallelic (dominant or X-linked hemizygous) or biallelic model, respectively.

To evaluated impact of rare coding variants on gene function, we used the “probability of loss-of-function intolerance” (pLI) score^15,16^ to rank the protein truncating variants (PTVs, including stopgain, stoploss, frameshift and canonical splice site variants) in 3 bins of severity (0.995–1, 0.5–0.995, 0–0.5) and the integrated “missense badness, PolyPhen-2, constraint” (MPC) score^17^, to rank the missense variants in other 3 bins of severity (MPC ≥ 2 (DmisB), 2 > MPC ≥ 1 (DmisA), 1 > MPC ≥ 0).

Genes previously associated with ASD or other NDDs were retrieved from gene lists compiled from two recent large ES and GS studies on ASD/NDD^5,6^ and from “GeneTrek” (https://genetrek.pasteur.fr/), a website centralizing NDD gene lists from the most relevant databases. All the *de novo* variants DNVs of interest were visually inspected using IGV^57^. *De novo* PTVs in constrained genes (PTV_0.5_) and other selected *de novo* missense variants were also validated by Sanger sequencing.

### Polygenic risk score

We calculated Polygenic Risk Scores (PRS) in 384 individuals of our cohort (European and Admixed ancestry), with GS data available. We used the additive model implemented in PLINK v.1.9^51^ and the summary statistics from a large ASD genome-wide association study (GWAS) ^2^. Prior to these analyses, we performed standard quality control steps^58^. Briefly, we excluded variants with MAF ≤ 0.01 or imputation INFO score ≤ 0.8 and ambiguous SNPs to avoid potential strand conflicts. Clumping was performed using an r2 threshold of 0.1 and a radius of 250 kb. Only SNPs with a *p*-value ≤ 0.1 were used for PRS computation. Each genotype was weighted with the variant’s OR and all the weighted variants were summed together into a PRS. The polygenic Transmission Disequilibrium Test (pTDT) was performed as previously described^42^. We evaluated pTDT test statistic as a two-sided, one-sample t test.

#### CNV data analysis.

For CNV identification we used two types of data: the Illumina Infinium PsychArray SNP data (available for the whole cohort, n = 435 individuals) and WGS data (available for 105 families, n = 392 individuals).

For SNP-array data, CNVs were identified as previously described^59^. Briefly, we used three different calling algorithms: PennCNV^60^, QuantiSNP^61^ and CNVPartition (Illumina). We then generated a stringent set of CNVs, defined as those called by ≥ 2 algorithms (with one being PennCNV) with moderately high confidence (confidence scores ≥ 10, CNV size ≥ 1Kb and number of consecutive probes for CNV detection ≥ 3) and with a reciprocal overlap at least 50%. If the CNV boundaries varied between the different calling algorithms, we retained the largest ones. CNVs were annotated for size, overlapping genes according to RefSeq, exonic content, overlap with segmental duplications, overlap with centromeric regions, overlap with known recurrent CNVs associated to neurodevelopmental disorders (defined as having at least 40% overlap with the loci reported in Table S3 of Douard et al.^62^), CNVs displayed in DGV, frequency in the stringent CNV list in our 230 ASD parents, overlap with copy-number stable regions according to the stringent CNV map of the human genome^63^, and NDD gene classification according to the “GeneTrek” database. After the annotation, to reduce false-positive calls, we retained only CNVs ≥ 10 kb in length and including ≥ 5 consecutive probes. Moreover, we selected only CNVs including at least one RefSeq exon (exonic CNVs). The trio option of PennCNV was used to confirm inheritance status of the resulting CNV calls. All *de novo* rare CNVs were manually curated by visual inspection of the BAF and LRR plots and false positives were excluded.

For WGS data, CNVs ≥ 10 kb were detected using Canvas^64^, a read-depth based CNV calling tool developed by Illumina. Rare CNVs were annotated and defined with the same criteria used for microarray data. To determine the CNV inheritance status, parents and child calls were compared and all overlapping calls were identified. CNV calls in the child with no overlapping CNVs in the parents were tagged as being potentially *de novo*. If some overlapping calls were found in a parent, but with a reciprocal overlap < 50% or the CNV type (deletion or duplication) did not match, then the CRAM files were manually inspected to visualize the CNV region in IGV.

To resolve the structures of duplications, determine the breakpoint coordinates of selected CNVs at the base level, and understand their impact on genes, we focused on rare CNVs that overlapped exons of NDD genes reported as HC by GeneTrek. CNV calls were analysed by manual inspection of paired-end reads and split reads at the breakpoint junctions visualizing CRAM files using IGV. By using IGV color coding to flag anomalous insert sizes and pair orientations, we were able to detect deletion and duplications and to classify duplications as tandem or inverted. Duplications were considered to possibly increase gene dosage when at least one RefSeq isoform was fully contained within the duplication. The effects of partial deletions, intragenic duplications and fusion genes created at the CNV breakpoints were assessed using the CCDS track of UCSC Genome Browser.

Finally, we analysed the overlap between SNP-array and GS CNV calls. After selecting CNVs ≥ 10 kb in length and including at least one exon, we restricted our analyses to rare CNVs defined as either known recurrent CNVs associated to NDDs or non-recurrent CNVs with the following characteristics: (i) having an overlap with segmental duplication or centromeric regions < 50%; (ii) having a frequency ≤ 1% in the 230 parents, using the 50% reciprocal overlap criteria; (iii) having more than 75% overlap with copy-number stable regions, according to the stringent CNV map of the human genome^63^. CNVs identified by both methods were considered high-quality calls and were retained with the coordinates obtained by CANVAS, refined by subsequent manual inspection. CNVs deemed relevant to NDDs identified by WGS but missed by SNP-array for lack of probes in the region or identified only by SNP-array in samples without WGS data, were experimentally validated by Sanger Sequencing and SYBR^®^ Green-based real-time quantitative PCR assays^59^.

## Figures and Tables

**Table 1:** List of *de novo* pdSNVs identified in affected individuals.

Variant location ^1^	ACMG class ^2^	Individual (Family structure) ^3^	Symptoms Onset Pattern; ADOS-2 (Diagnosis, comparison score); CARS2-ST score; Language level; ID level (test, score); Epilepsy ^4^
**a) PTV**_**0.5**_			
NM_002884.4(RAP1A): c.73C > T(p.Q25X)	P (PVS1,PS2,PM2,PP3)	69.3 (f/m/aM)	EO; ADOS-2 (Autism, 9); CARS2-ST: 41.5; Absent speech; Moderate ID (NA); Focal epilepsy
NM_170692.4(RASAL2): c.2560C > T(p.Q854X)	LP (PS2,PM2,PP3)	19.3 (f/m/aM)	EO; ADOS-2 (Autism, 6); CARS2-ST: 42.5; Absent speech; Mild ID (NA)
**NM_006922.4(SCN3A): c.4888C > T (p.R1630X)**	**LP (PS2,PM2,PP3,BS4)**	**40.3 (f/m/**aM**/aM)**	**Mixed; ADOS-2 (Autism, 9); CARS2-ST: 37.5; Single words; Normal IQ (Leiter-R, B-IQ 117, FR 108)**
**NM_001040142.2(SCN2A): c.4180C > T (p.Q1394X)**	**P (PVS1,PS2,PM2,PP3,BS4)**	**113.4 (f/m/aF/**raM**/uF)**	**EO; ADOS-2 (Autism, 10); CARS2-ST: 43; Absent speech; Severe ID (NA)**
NM_015902.6(UBR5): c.7441del (p.H2481Mfs*7)	**LP (PVS1,PS2,PM2,BS4)**	**110.3 (f/m/**aM**/aM/uMs)**	**EO; ADOS-2 (Autism, 6); CARS2-ST: 37; Atypical language; Borderline IQ (Leiter-R, B-IQ 74)**
NM_001256627.2(BRSK2): c.1620_1621del (p.D540Efs*9)	**P (PVS1,PS2,PM2)**	**32.3 (f/m/**aM**/uM)**	**EO; ADOS-2 (Autism, 6); CARS2-ST: 39.5; Single words; Normal IQ (Leiter-R, B-IQ 96, FR 108)**
NM_005853.6(IRX5): c.28C > T (p.Q10X)	P (PVS1,PS2,PM2,PP3)	102.3 (f/m/aM)	EO; ADOS-2 (Autism, 10); CARS2-ST: 39; Atypical language; Borderline IQ (NA)
**NM_001372044.2(SHANK3):c.2611G > T (p.E871X)**	**P (PVS1,PM2,PM6, PP3)**	**123.3** **(f/**aF**)** ^5^	**EO; ADOS-2 (Autism, 9); CARS2-ST: 43.5; Absent speech; Severe ID (NA)**
**NM_001372044.2(SHANK3):c.4871C > A (p.S1624X)**	**LP (PS2,PM2,PP3)**	**29.3 (f/m/**aF**/uM)**	**EO; ADOS-2 (Autism, 8); CARS2-ST: 43; Absent speech; Profound ID (GMDS 0–2, CA 11 y, total AE 12,75 m); Focal epilepsy**
**b) DmisB**			
NM_003047.5(SLC9A1): c.1172A > G (p.E391G)	LP (PS2,PM1,PM2,PP2)	98.3 (f/m/aM)	EO; ADOS-2 (Autism, 10); CARS2-ST: 42; Single words; Borderline IQ (Leiter-R, B-IQ 76, FR 84)
NM_014263.4(YME1L1): c.1981G > A (p.E661K)	LP (PS2,PM1,PM2,PP3,BS4)	117.3 (f/m/aM/aF)	EO; ADOS-2 (Autism, 9); CARS2-ST: 35.5; Atypical language; Mild ID (WPPSI-III, V-IQ 80, P-IQ 65, Total IQ 66); Focal epilepsy
**NM_001256627.2(BRSK2): c.472G > A (p.A158T)** ^6^	**LP (PS2,PM1,PM2,BS4)**	**14.4 (f/m/aM/**aM**)**	**EO; ADOS-2 (Autism, 8); CARS2-ST: 39; Absent speech; Moderate ID (NA)**
**NM_002804.5(PSMC3): c.1180G > A (p.A394T)**	**LP (PS2,PM1,PM2,PP3)**	**106.3 (f/m/**aM**)**	**EO; ADOS-2 (Autism, 9); CARS2-ST: 42.5; Absent speech; Severe ID (NA)**
**NM_001376.5(DYNC1H1): c.7501T > C (p.S2501P)**	**LP (PS2,PM2)**	**122.3 (f/m/**aM**)**	**EO; ADOS-2 (Autism, 9); CARS2-ST: 36; Single words; Normal IQ (NA)**
NM_016530.3(RAB8B): c.227C > T (p.A76V) ^7^	LP (PS2,PM1,PM2,PP3,BS4)	85.3 (f/m/aM/aM)	Mixed; ADOS-2 (Autism, 7); CARS2-ST: 45; Absent speech; Moderate ID (NA)
NM_001199172.2(MGAT5B):c.1177G > A (p.D393N) ^7^	LP (PS2,PM1,PM2,PP3,BS4)	15.3 (f/m/aM/aM)	EO; ADOS-2 (Autism, 9); CARS2-ST: 42.5; Single words; Normal IQ (Leiter-R, B-IQ 89, FR 84)
**NM_004218.4(RAB11B): c.98G > A (p.R33H)**	**LP (PS2,PM1,PM2,PP2)**	**52.3 (f/m/**aM**)**	**EO; ADOS-2 (Autism, 6); CARS2-ST: 39; Single words; Mild ID (Leiter-R, B-IQ 56, FR 63); Focal epilepsy**
**NM_001365902.3(NFIX): c.224T > C (p.L75P)**	**LP (PS2,PM1,PM2,PP3,BP1)**	**81.3 (f/m/**aM**/uM)**	**EO; ADOS-2 (Autism, 10); CARS2-ST: 39.5; Absent speech; Severe ID (GMDS 0–2, CA 34 m, total AE 10 m)**
NM_020132.5(AGPAT3): c.536G > A (p.R179H)	LP (PS2,PM1,PM2,PP3)	17.3 (f/m/aM/uM)	Regressive; ADOS-2 (Autism, 9); CARS2-ST: 37; Single words; Mild ID (Leiter-R, Full IQ 57, FR 48)
NM_002409.5(MGAT3): c.1403C > T (p.T468M) ^7^	LP (PS2,PM2)	5.3 (f/m/aM)	EO; ADOS-2 (Autism, 5); CARS2-ST: 39.5; Single words; Borderline IQ (Leiter-R, B-IQ 79, FR 62)
**c) DmisA**			
NM_152365.3(KDF1): c.1058C > T (p.S353F)	LP (PS2,PM2,PP2)	81.3 (f/m/aM/uM)	EO; ADOS-2 (Autism, 10); CARS2-ST: 39.5; Absent speech; Severe ID (GMDS 0–2, CA 34 m, total AE 10 m)
NM_138417.3(KTI12): c.176G > A (p.R59H)	LP (PS2,PM2,PP3,BS4)	16.4 (f/m/aM/aM)	EO; ADOS-2 (Autism, 9); CARS2-ST: 39; Absent speech; Moderate ID (PEP-3, CA 2.7 y, cognitive-verbal AE < 12 m)
**NM_001348768.2(HECW2): c.2426T > C (p.L809P)**	**P (PS2,PM1,PM2,PP2,PP3)**	**68.3 (f/m/**aF**/uF/uM)**	**Mixed; ADOS-2 (Autism, 10); CARS2-ST: 38.5; Absent speech; Borderline IQ (Leiter-R, B-IQ 74, FR 73)**
NM_152783.5(D2HGDH): c.376G > A (p.V126M) ^7^	LP (PS2,PM1,PM2,PP3)	87.3 (f/m/aM/uM)	EO; ADOS-2 (Autism, 6); CARS2-ST: 40.5; Single words; Mild ID (Bayley-III, CA 38 m, AE 19 m)
**NM_001967.4(EIF4A2): c.727A > G (p.T243A)**	**LP (PS2,PM1,PM2,BS4)**	**110.4 (f/m/aM/**aM**/uMs)**	**EO; ADOS-2 (ASD, 4); CARS2-ST: 33; Atypical language; Normal IQ (Leiter-R, B-IQ 107, FR 102)**
NM_001010852.4(CLVS2): c.142C > G (p.R48G)	LP (PS2,PM1,PM2)	44.3 (f/m/aM/uF)	EO; ADOS-2 (Autism, 7); CARS2-ST: 45; Single words; Mild ID (Leiter-R, B-IQ 54, FR 52)
**NM_000368.5(TSC1): c.2023G > T (p.D675Y)**	**LP (PS2,PM1,PM2,PP3)**	**100.3 (f/m/**aF**)**	**EO; ADOS-2 (Autism, 9); CARS2-ST: 40; Single words; Mild ID (NA)**
NM_182765.6(HECTD2): c.742G > A (p.V248I)	LP (PS2,PM1,PM2)	30.3 (f/m/aM/uM)	EO; ADOS-2 (Autism, 9); CARS2-ST: 41.5; Absent speech; Moderate ID (PEP-3, CA 3 y, cognitive-verbal AE 17 m)
NM_020123.4(TM9SF3): c.941T > C (p.I314T)	LP (PS2,PM2,BS4)	12.5 (f/m/aM/uM/aM)	EO; ADOS-2 (Autism, 6); CARS2-ST: 33.5; Single words; Mild ID (GMDS 0–2, CA 17 m, total AE 11 m)
**NM_032138.7(KBTBD7): c.1849C > T (p.R617C)** ^7^	**VUS (PS2,PP3,BS4)**	**40.4 (f/m/aM/**aM**)**	**Mixed; ADOS-2 (Autism, 8); CARS2-ST: 34.5; Atypical language; Normal IQ (Leiter-R, B-IQ 111, FR 88)**
NM_021239.3(RBM25): c.1052G > A (p.R351H) ^7^	LP (PS2,PM1,PM2)	60.3 (f/m/aF)	EO; ADOS-2 (Autism, 10); CARS2-ST: 48; Single words; Moderate ID (PEP-3, CA 5.6 y, cognitive-verbal AE 36m)
NM_001017919.2(RCCD1): c.419C > A (p.A140D)	VUS (PS2,PM1,PM2,BS4,BP4)	117.4 (f/m/aM/aF)	EO; ADOS-2 (Autism, 8); CARS2-ST: 34.5; Single words; Normal IQ (Leiter-3, IQ 97)
**NM_000430.4(PAFAH1B1): c.431G > A (p.R144Q)**	**LP (PS2,PM1,PM2,BS4)**	**15.4 (f/m/aM/**aM**)**	**EO; ADOS-2 (Autism, 6); CARS2-ST: 33; Normal language; Borderline IQ (WISC-IV, Full IQ 78)**
NM_032442.3(NEURL4): c.2170G > A (p.G724S) ^7^	LP (PS2,PM1,PM2,PP3,BS4)	115.3 (f/m/aF/aM/aF)	EO; ADOS-2 (Autism, 6); CARS2-ST: 36; Single words; Moderate ID (GMDS, CA 27 m, total AE 5.5 m)
NM_014738.6(TMEM94): c.3202C > T (p.R1068C) ^7^	LP (PS2,PM1,BP1)	101.3 (f/m/aM)	EO; ADOS-2 (ASD, 4); CARS2-ST: 36.5; Absent speech; Moderate ID (GMDS, CA 43 m, total AE 21.5 m)
NM_017534.6(MYH2): c.3458G > A (p.S1153N)	LP (PS2,PM1,PM2,PP3,BS4)	77.3 (f/m/aM/aF/uM/uFs)	EO; ADOS-2 (Autism, 9); CARS2-ST: 41.5; Single words; Mild ID (Leiter-R, NA)
NM_007050.6(PTPRT): c.1076G > A (p.R359Q) ^7^	LP (PS2,PM1)	72.3 (f/m/aF/uF)	EO; ADOS-2 (Autism, 6); CARS2-ST: 34; Single words; Mild ID (Leiter-R, NA)
NM_017436.7(A4GALT): c.562G > A (p.G188S) ^7^	LP (PS2,PM1,PM2,PP3)	90.3 (f/m/aF/dM)	EO; ADOS-2 (Autism, 9); CARS2-ST: 41; Absent speech; Moderate ID (PEP-3, CA 4 y, cognitive-verbal AE 24 m)

*De novo* pdSNVs include PTVs in genes with pLI score ≥0.5 (PTV_0.5_), missense variants with MPC score ≥2 (DmisB) and missense variants with MPC score 1–2 (DmisA). All reported variants are novel, except for variants labelled with ^6^ and ^7^.

1 Amino acid changes are reported according to the MANE isoform; Variant in high-confidence ASD/NDD gene list classification according to Table S6 are indicated in bold

2 Classification of genetic variants according to the ACMG guidelines, together with detailed evidence codes, was performed using InterVar. *Abbreviations*: P, pathogenic; LP, likely pathogenic; VUS, variant of uncertain significance

3 Probands are indicated with an identifier code formed by the family number and the individual number. In brackets, the family structure is reported with children listed sequentially after the father and the mother, according to the recruitment order. The affected individual heterozygote for the variant is underlined. *Abbreviations*: f, father; m, mother; r, relative (uncle); a, affected; u, unaffected; M, male; F, female.

4 Epilepsy is present only where it is explicitly indicated. Abbreviations: EO, Early Onset; NA, Not Assessed; V-IQ, Verbal IQ; P-IQ, Performance IQ; B-IQ, Brief IQ; FR, Fluid Reasoning; CA, chronological age; AE, age equivalent; GMDS, Griffiths Mental Development Scales; m, months; y, years.

5 Since DNA of the biological mother was unavailable, *de novo* status was only assumed.

6 This variant has been reported in gnomAD v3.1.2 in a single individual of African/African American origin belonging to the gnomAD “neuro” dataset, therefore the variant is not present in gnomAD v3.1.2 “non-neuro” dataset.

7 Not novel variant, present in gnomAD v3.1.2 “non-neuro” dataset with MAF ≤0.01%.

**Table 2:** Potentially damaging CNVs (pdCNVs) identified in our cohort.

CytoBand	Coordinates (hg38)	Detection	Individual (Family)	RefSeq Genes ^1, 2^	ACMG
**a) Large CNVs**					
18p11.22-p11.21	NC_000018.10:g.(9291609_12509914)_(12509915_15728221)dup	SNP/WGS^5^	9.4 (f/m/aM/dM/uF)	31 genes[**GNAL**; **PPP4R1**; **RALBP1**]	LP
**b) Recurrent Genomic Loci (RGD)**					
2p16.3	NC_000002.12:g.(49962600_50756048)del	SNP^6^	2.3 (f/m/aF)	2 genes[**NRXN1**]	LP
15q11.2	NC_000015.10:g.(22636123_23102073)_(23102074_23568025)dup	SNP/WGS	99.4 (f/m/aM/aM)	6 genes[**CYFIP1**]	VUS
15q11.2	NC_000015.10:g.(22636123_23117882)del	SNP^6^	117.3 (f/m/aM/aF)	6 genes[**CYFIP1**; **NIPA2**]	LP
15q13.2-q13.3	NC_000015.10:g.(30626840_32222140)del	SNP^6^	115.3 (f/m/aF/aM/aF)	12 genes[**CHRNA7**; **OTUD7A**]	LP
15q13.2-q13.3	NC_000015.10:g.(30635159_32222140)_(32222141_33809123)dup	SNP/WGS	81.3 (f/m/aM/uM)	12 genes[**OTUD7A**]	VUS
16p11.2	NC_000016.10:g.(29584162_30188392)del	SNP^6^	118.4 (f/m/aM/aF/uM)	32 genes[**TAOK2**;**CORO1A**;**MAZ**;**PRRT2**]	P
**c) de novo CNVs** ^4^					
5q21.3	NC_000005.10:g.(108496181_109233820)_(109233821_109971461)dup	SNP/WGS^5^	73.3 (f/m/aF/dM)	*FER,LINC01023*	VUS
2p16.2	NC_000002.12:g.(54193480_54356405)del	SNP^6,8^	120.4 (f/m/aM/aM**)**	*ACYP2,C2orf73,TSPYL6*	VUS
**d) CNVs including dosage-sensitive NDD genes**					
1p36.13	NC_000001.11:g.(18606250_19018007)del	SNP^6^	91.3 (m/aM/dM/aM/dM)	6 genes[**ALDH4A1**,**PAX7**]	VUS
1p31.1	NC_000001.11:g.(71519763_71888101)del	SNP/WGS^8^	108.3 (f/m/aM)	**NEGR1**,*NEGR1-IT1*	VUS
1q42.13	NC_000001.11:g.(230095012_230188321)_(230188322_230281632)dup	SNP^6^	2.3 (f/m/aF)	**GALNT2** ^9^	VUS
1q44	NC_000001.11:g.(245334797_245424055)del	SNP/WGS	71.3 (f/m/aF)	**KIF26B**	VUS
2p25.3	NC_000002.12:g.(196082_261610)del	SNP/WGS	100.3 (f/m/aF)	**SH3YL1**	VUS
2p25.3	NC_000002.12:g.(1736572_1846326)_(1846327_1956082)dup	WGS^7^	84.3 (f/m/aF)	*MYT1L,PXDN* ^10^	VUS
2p25.2	NC_000002.12:g.(6833046_6845996)del	WGS^7^	113.4 (f/m/aF/raM/uF)	**CMPK2**,*NRIR*	VUS
4q31.3	NC_000004.12:g.(150267053_150327676)_(150327677_150388301)dup	SNP/WGS	11.3 (f/m/aM/uM)	**LRBA** ^9^	VUS
5q14.1	NC_000005.10:g.(79401623_79420576)del	WGS^5,7^	82.3 (f/m/aM/uF)	**HOMER1**	VUS
6q12	NC_000006.12:g.(63563200_63698372)del	SNP^6,8^	117.3 (f/m/aM/aF)	**PHF3**,**PTP4A1**,*LOC128125822*	VUS
6q25.3	NC_000006.12:g.(156870642_156885949)del	SNP/WGS	56.3 (f/m/aF/aM)	**ARID1B**	VUS
7q32.1	NC_000007.14:g.(127719389_127968829)del	SNP/WGS	109.3 (f/m/aM)	**SND1**	VUS
8q22.3	NC_000008.11:g.(104392687_104406426)del	SNP/WGS	85.3 (f/m/aM/aM)	**DPYS**	VUS
9p24.2	NC_000009.12:g.(2726669_2792829)del	SNP/WGS	14.3 (f/m/aM/aM)	**KCNV2**	VUS
9p21.1-p13.3	NC_000009.12:g.(33140780_33262543)_(33262544_33384308)dup	SNP/WGS	46.3 (f/m/aM/uM)	*BAG1,B4GALT1,B4GALT1-AS1,SPINK4* ^10^	VUS
9q22.32	NC_000009.12:g.(95221201_95255683)del	SNP/WGS	96.3 (f/m/aM/uF)	**FANCC**	VUS
10p15.2	NC_000010.11:g.(3040783_3121006)del	SNP/WGS	17.3 (f/m/aM/uM)	**PFKP**,*PFKP-DT*	VUS
10q24.1	NC_000010.11:g.(95440563_95689410)_(95689411_95938259)dup	SNP/WGS	47.3 (f/m/aM)	**ALDH18A1**,*SORBS1,TCTN3* ^10^	VUS
10q24.32	NC_000010.11:g.(102640823_102666335)_(102666336_102691849)dup	WGS^7^	67.3 (f/m/aM/uF)	**TRIM8**	VUS
10q25.2-q25.3	NC_000010.11:g.(113078246_113111305)del	WGS^7^	47.3 (f/m/aM)	**TCF7L2**	VUS
13q31.3	NC_000013.11:g.(93533137_93587893 )_(93587894_93642651)dup	SNP/WGS	94.3 (f/m/aM)	**GPC6** ^9^	VUS
16p13.2	NC_000016.10:g.(8446703_8794331)del	SNP/WGS	105.3 (f/m/aM)	**ABAT**,*TMEM114,METTL22*	VUS
16q23.1	NC_000016.10:g.(78256339_78314555)del	SNP/WGS	45.3 (f/m/aM/uF)	**WWOX**	VUS
20q13.33	NC_000020.11:g.(62821525_62833156)del	SNP/WGS	96.3 (f/m/aM/uF)	**COL9A3**	VUS
21q22.11	NC_000021.9:g.(31165520_31653872)del	SNP/WGS	55.3 (f/m/aM/aM/dM)	**TIAM1**,*TIAM1-AS1*	VUS
21q22.3	NC_000021.9:g.(46140262_46168679)del	SNP/WGS	64.3 (f/m/aM)	**FTCD**,*FTCD-AS1,SPATC1L*	VUS

The CNVs reported in this table are divided according to the 4 categories used for the prioritization: variants larger than 3 Mb, variants in known recurrent genomic loci (RGD), *de novo* variants, variants overlapping dosage-sensitive GeneTrek genes (https://genetrek.pasteur.fr/). GeneTrek classification for genes included in CNVs is reported in Table S6. Dosage-sensitive genes are defined according to pLI, pHaplo and pTriplo gene scores (pLI^3^0.5 and/or pHaplo^3^0.55 for genes in CNVs potentially disrupting the CDS, pTriplo^3^0.68 for duplications including the entire CDS of genes). Probands are indicated with an identifier code formed by the family number and the individual number. In the segregation column, children are listed sequentially after the father and the mother, according to recruitment order. Family members carrying the CNV are underlined; if the CNV is a *de novo* variant, the heterozygote is also indicated in bold. *Abbreviations*: a, affected; u, unaffected; d, specific learning disability; M, male; F, female; f, father; m, mother; r, relative (uncle); SNP, SNP data analysis; WGS, genome sequencing data analysis.

*Symbols*:

1 GeneTrek dosage-sensitive genes according to pLI/pHaplo or pTriplo scores are indicated in bold

2 If more than 5 genes are affected by the CNV, only dosage-sensitive GeneTrek genes are listed (in brackets)

3 Classification of genetic variants according to the ACMG guidelines*; Abbreviations*: P, pathogenic; LP, likely pathogenic; VUS, variant of uncertain significance

4 *De novo* CNVs not included in previous categories (a and b)

5 CNV validated by Sanger sequencing

6 CNV detected only from SNP data because WGS was not carried out for the family

7 CNV detected only from WGS data

8 CNV validated by qPCR

9 Possible intragenic duplication of haplo-insufficient GeneTrek genes

10 Possible gene fusion including GeneTrek dosage-sensitive genes.

a) Large CNVs (≥ 3 Mb). Probands with large CNVs were not present in our cohort, because most of them had been previously screened by array-CGH in a clinical setting. However, we identified a 3.2 Mb *de novo* tandem duplication of chr18p11 in one SLD sibling diagnosed with language and learning delay (Fig. S6).

b) Recurrent CNVs. This category included 4 deletions and 2 duplications consistent with known RGD.

c) De novo CNVs. In addition to the two *de novo* CNVs included in the previous categories, we identified a 5q21.3 tandem duplication including the entire *FER* gene and a 2p16.2 deletion affecting the brain expressed gene *ACYP2*.

d) Rare CNVs affecting dosage-sensitive NDD genes reported in GeneTrek^19^. This category included 19 deletions and 7 duplications, selected among deletions or intragenic duplications potentially disrupting the CDS of genes with pHaplo ≥ 0.55^20^ and/or pLI ≥ 0.5, duplications involving the whole CDS of genes with pTriplo ≥ 0.68^20^ and CNVs potentially leading to in frame fusion transcripts. Among these, the inherited deletions involving *PHF3*, *NEGR1, HOMER1* and *TIAM1* are of particular interest, as these neurodevelopmental genes have been previously implicated in ASD/NDD.

## Data Availability

The genomic and phenotypic data for the families analysed in this study are available by request from dbGAP (dbGaP accession phs002509.v1.p1).
